# A Secure Semi-Field System for the Study of *Aedes aegypti*

**DOI:** 10.1371/journal.pntd.0000988

**Published:** 2011-03-22

**Authors:** Scott A. Ritchie, Petrina H. Johnson, Anthony J. Freeman, Robin G. Odell, Neal Graham, Paul A. DeJong, Graeme W. Standfield, Richard W. Sale, Scott L. O'Neill

**Affiliations:** 1 School of Public Health, Tropical Medicine and Rehabilitation Sciences, James Cook University, Cairns, Queensland, Australia; 2 About Awnings, Gordonvale, Queensland, Australia; 3 Power, Graham and Dempsey Pty Ltd Architects, Cairns, Queensland, Australia; 4 Hutchisons Builders Pty Ltd, Smithfield, Queensland, Australia; 5 MGF Consultants (NQ) Pty Ltd, Cairns, Queensland, Australia; 6 S2F Pty Ltd, Brisbane, Queensland, Australia; 7 School of Biological Sciences, The University of Queensland, St Lucia, Queensland, Australia; Duke University-National University of Singapore, Singapore

## Abstract

**Background:**

New contained semi-field cages are being developed and used to test novel vector control strategies of dengue and malaria vectors. We herein describe a new Quarantine Insectary Level-2 (QIC-2) laboratory and field cages (James Cook University Mosquito Research Facility Semi-Field System; MRF SFS) that are being used to measure the impact of the endosymbiont *Wolbachia pipientis* on populations of *Aedes aegypti* in Cairns Australia.

**Methodology/Principal Findings:**

The MRF consists of a single QIC-2 laboratory/insectary that connects through a central corridor to two identical QIC-2 semi-field cages. The semi-field cages are constructed of two layers of 0.25 mm stainless steel wire mesh to prevent escape of mosquitoes and ingress of other insects. The cages are covered by an aluminum security mesh to prevent penetration of the cages by branches and other missiles in the advent of a tropical cyclone. Parts of the cage are protected from UV light and rainfall by 90% shade cloth and a vinyl cover. A wooden structure simulating the understory of a Queenslander-style house is also situated at one end of each cage. The remainder of the internal aspect of the cage is covered with mulch and potted plants to emulate a typical yard. An air conditioning system comprised of two external ACs that feed cooled, moistened air into the cage units. The air is released from the central ceiling beam from a long cloth tube that disperses the airflow and also prevents mosquitoes from escaping the cage via the AC system. Sensors located inside and outside the cage monitor ambient temperature and relative humidity, with AC controlled to match ambient conditions. Data loggers set in the cages and outside found a <2°C temperature difference. Additional security features include air curtains over exit doors, sticky traps to monitor for escaping mosquitoes between layers of the mesh, a lockable vestibule leading from the connecting corridor to the cage and from inside to outside of the insectary, and screened (0.25 mm mesh) drains within the insectary and the cage. A set of standard operating procedures (SOP) has been developed to ensure that security is maintained and for enhanced surveillance for escaping mosquitoes on the JCU campus where the MRF is located. A cohort of male and female *Aedes aegypti* mosquitoes were released in the cage and sampled every 3–4 days to determine daily survival within the cage; log linear regression from BG-sentinel trapping collections produced an estimated daily survival of 0.93 and 0.78 for females and males, respectively.

**Conclusions/Significance:**

The MRF SFS allows us to test novel control strategies within a secure, contained environment. The air-conditioning system maintains conditions within the MRF cages comparable to outside ambient conditions. This cage provides a realistic transitional platform between the laboratory and the field in which to test novel control measures on quarantine level insects.

## Author Summary

Novel vector control strategies require validation in the field before they can be widely accepted. Semi-field system (SFS) containment facilities are an intermediate step between laboratory and field trials that offer a safe, controlled environment that replicates field conditions. We developed a SFS laboratory and cage complex that simulates an urban house and yard, which is the primary habitat for *Aedes aegypti*, the mosquito vector of dengue in Cairns Australia. The SFS consists of a Quarantine Insectary Level-2 (QIC-2) laboratory, containing 3 constant temperature rooms, that is connected to two QIS-2 cages for housing released mosquitoes. Each cage contains the understory of a “Queenslander” timber house and associated yard. An automated air conditioning system keeps temperature and humidity to within 1°C and 5% RH of ambient conditions, respectively. Survival of released *A. aegypti* was high, especially for females. We are currently using the SFS to investigate the invasion of strains of *Wolbachia* within populations of *A. aegypti*.

## Introduction

Dengue is the most abundant arboviral infection in the tropics, with 50–100 million cases and 5 million people at risk annually [1]. Currently, there is no available human vaccine, thus dengue prevention is limited to control strategies attacking the mosquito vectors *Aedes aegypti* and *Aedes albopictus*. Outside of community education programs and source reduction campaigns that seek to remove artificial containers that produce the vectors, most government programs rely upon insecticides to reduce vector populations. These methods are often inefficient, costly and ineffective. Furthermore, many populations of *A. aegypti* have developed physiological resistance to many pesticides, rendering them ineffective [2].

Thus, novel population control strategies are being developed to control vectors of dengue and other mosquito-borne diseases. Releases of genetically modified (GM) *A. aegypti* that are refractory to dengue infection and transmission, and the inundative release of sterile males are currently being developed to reduce populations of *A. aegypti* [3], [4]. Our research group is investigating the use of strains of the endosymbiotic bacteria *Wolbachia pipientis* to induce life-shortening and dengue virus interference in populations of *A. aegypti*. The *Wolbachia* infection is driven to fixation in populations of *A. aegypti* via a cytoplasmic incompatibility mechanism. The *w*MelPop strain is known to shorten the life span of *A. aegypti* reducing the number of individuals that survive long enough to transmit dengue [5], and can also interfere with the replication and transmission of several viruses in *A. aegypti*, including DENV-2 and chikungunya virus [6]. Entomopathogenic fungi are also being studied for their life-shortening impact on *A. aegypti* [7].

Novel control strategies require confirmation under field conditions before they can be deployed operationally. Furthermore, experiments involving population replacement methods involving GM and novel agents such as *Wolbachia* and fungi that are conducted out of the laboratory must be under tight containment to avoid accidental release. A cross-disciplinary scientific working group developed guidelines for testing of gene drive systems within secure flight cages [8]. These facilities, termed “semi-field system” (SFS) [9], typically consist of secure biocontainment laboratory for insect rearing, secure field cage for experimental release, and associated security features such as fencing, moats and pass-coded gates. Within the cages, experimental houses or huts simulating domestic premises to be tested are featured. This is especially important for *A. aegypti*, a mosquito that typically feeds on humans and harbours within houses and other human premises. Natural substrates of soil, grass and native vegetation are included. Natural larval habitat such as puddles for *Anopheles* malaria vectors and artificial containers for *Aedes* are included. Biocontainment structures typically include double-door atriums, air curtains, mosquito surveillance traps, double layers of insect-proof screening, screened water drains, etc. In the tropics, facilities must often be built to withstand heavy rain and strong winds, often to tropical storm, cyclone or hurricane strength.

We describe a SFS that features a biocontainment level 2 laboratory/insectary that connects directly to 2 identical Quarantine Insectary Containment level 2 (QIC-2) semi-field cages. This new facility, the James Cook University Mosquito Research Facility (MRF), is currently being used to investigate the impact of *w*MelPop and *w*Mel strains of *Wolbachia* infection on survival of *A. aegypti*, and the dynamics of its spread within a population of wild type *A. aegypti*. Each of the SFS cages contains the ground floor of a simulated Queenslander house and associated yard. Queenslander houses are typically timber houses set on concrete or wooden pillars, and are common throughout much of Queensland, Australia (http://en.wikipedia.org/wiki/Queenslander_%28architecture%29). They are generally unscreened to maximise ventilation, and the ground floor is often not fully enclosed, allowing free access to mosquitoes. Dengue transmission is often most intense in suburbs dominated by these older types of housing [10]. We describe the security and containment features of the MRF, measure the environmental conditions inside and outside the cage and the impact of its climate control system, and also examine the survival of wild type *A. aegypti* within the cage. Finally, we provide standard operating procedures (SOP; File S1) designed to prevent escape of released mosquitoes.

## Materials and Methods

### Ethics statement

Human ethics approval for use of human volunteers to blood feed colony (dengue free) *A. aegypti* was obtained (JCU Human Ethics H2250). Volunteers were examined for fever before each blood feeding, excluded if feverish, and could withdraw at anytime. Written consent was obtained from all staff involved in blood feeding.

### Construction of the MRF insectary and semi-field system cages

**Figure 1:**
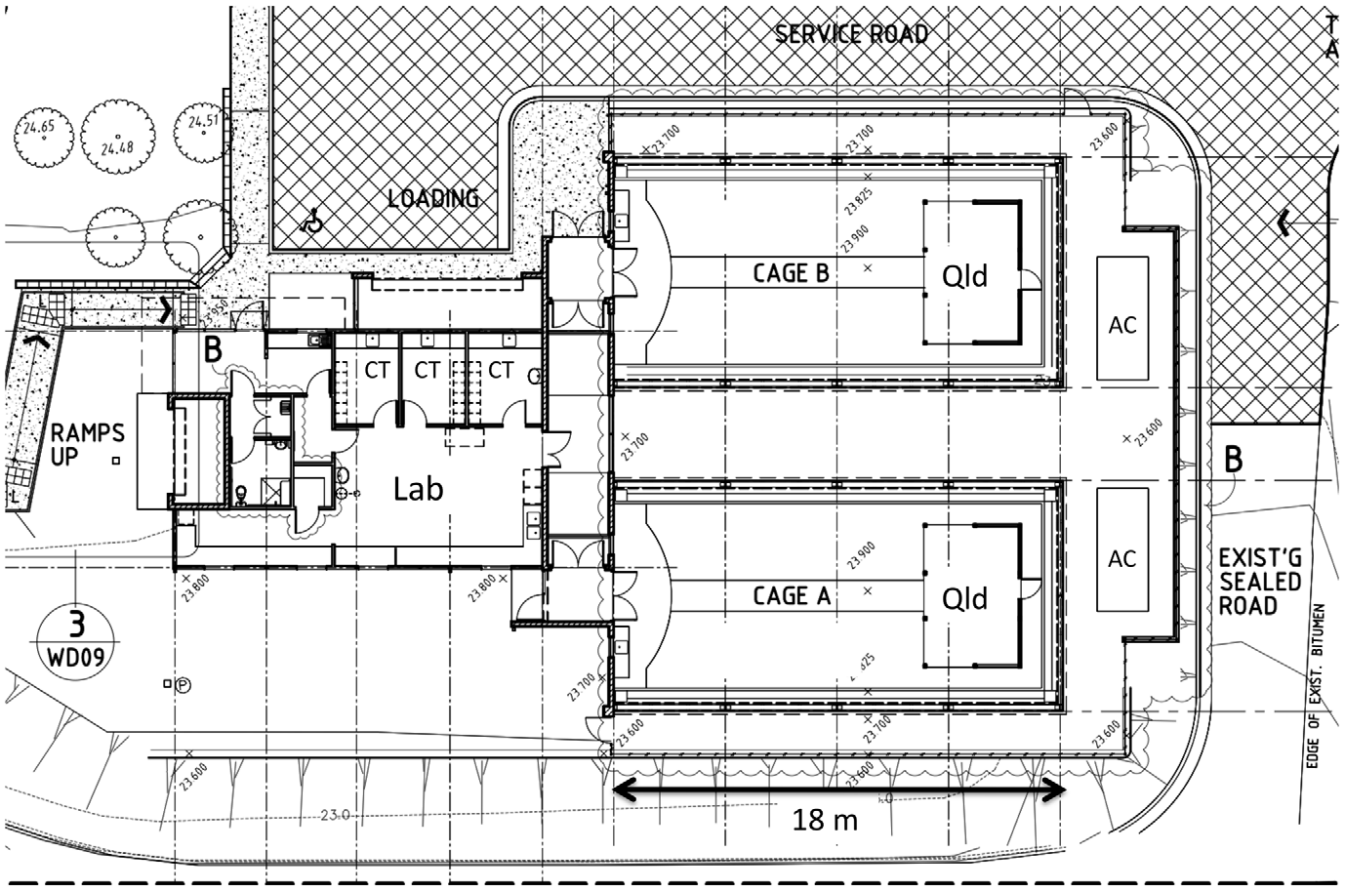
Architectural plan for the James Cook University Mosquito Research Facility semi-field system. The laboratory/insectary with constant temperatures rooms (CT) and SFS cages with simulated Queenslander (Qld) and air conditioning system (AC) shown.

The MRF was constructed to provide a simulated Cairns urban environment, under QIC-2 containment levels (http://www.daff.gov.au/aqis/import/general-info/qap/class7/quarantine_approved_criteria_qap_class_7.2_quarantine_insectary_containment_level_2_qic2_facilities), for testing novel control strategies on *A. aegypti*. The MRF is built on 133 m^2^ of land on the Smithfield campus of James Cook University (16°48′58”S, 145°41′15”E) located ca. 15 km northwest of the city of Cairns, Queensland Australia. Cairns is located in the wet tropics of northern Queensland, and has a pronounced wet and dry monsoonal climate; the mean daily temperature ranges from 21°C in winter to 27°C in summer, and an average of 1992 mm of rain falls annually (Australian Bureau of Meteorology; http://www.bom.gov.au/index.shtml). Cairns has a history of dengue outbreaks [10], [11], and *A. aegypti* are present in most urban areas. The campus building site was chosen as it is practical for researchers but, more importantly, it is situated within tropical rainforest and is isolated from urban areas of Cairns where *A. aegypti* is common. Thus, we think that any escaping *A. aegypti* are highly unlikely to breed with existing populations in the Cairns region. Construction on the MRF began in March 2008 and finished in January 2009. The cost of the facility in $AUS was $469,000 for the cage, $888,000 for the laboratory and $364,000 for the air conditioning system including controller. Total cost was $1,721,000; with Goods and Services Tax (10%) this was $1,893,000. Of this total, 55% was material costs, and 45% labor. The MRF design (Figure 1) allowed us to provide direct and secure access between the rearing laboratory and the SFS cages. Two cages were built so that treatment and control experiments could be conducted simultaneously. A service road connects to a loading bay located near the entry to the MRF laboratory.

#### Laboratory and semi-field system insectary

The MRF entrance leads to a common meeting room with a kitchen, sink, and an adjoining bathroom (Figure 1). From the meeting room, a double door vestibule with air curtain (positioned over meeting room) leads to a QIC-2 laboratory and mosquito rearing facilities. The main laboratory consists of a large open area with stainless steel benches for standard laboratory work. Three controlled temperature rooms (CT rooms) for rearing mosquitoes connect to the main laboratory. Temperature within the CT rooms could be set between 20–30°C but was maintained at 26°C for mosquito rearing. Humidity was not controlled centrally. Adult mosquito cages were enclosed in plastic wrap with damp sponges to maintain high humidity in each cage. The main laboratory then leads through an automatic locking door into the corridor connecting the laboratory to the SFCs. The corridor (internal dimensions 2.4 m wide×10.5 m long×2.7 m high excluding airlocks) is constructed of concrete block overlaid with plaster board that leads to two airlocked vestibules (2.4 m×2.2 m) that each connect to a cage (cage A and B). Entry into each cage requires activation of the door lock that prevents the concurrent opening of both vestibule doors. The laboratory, CT rooms, connecting corridor and vestibules have white walls and floors to enhance visual location of free-flying and resting mosquitoes. The main entry vestibule is also fitted with a long wall mirror so that staff can inspect for mosquitoes behind themselves. A locked emergency exit door was also present in each cage entry vestibule. The doorway into the cage was fitted with a vertical doorway (on the cage side) and a fine polyester mesh curtain (on the vestibule side) to minimise entry of mosquitoes from the cage when the door is open.

#### The MRF SFS cages: structural

**Figure 2:**
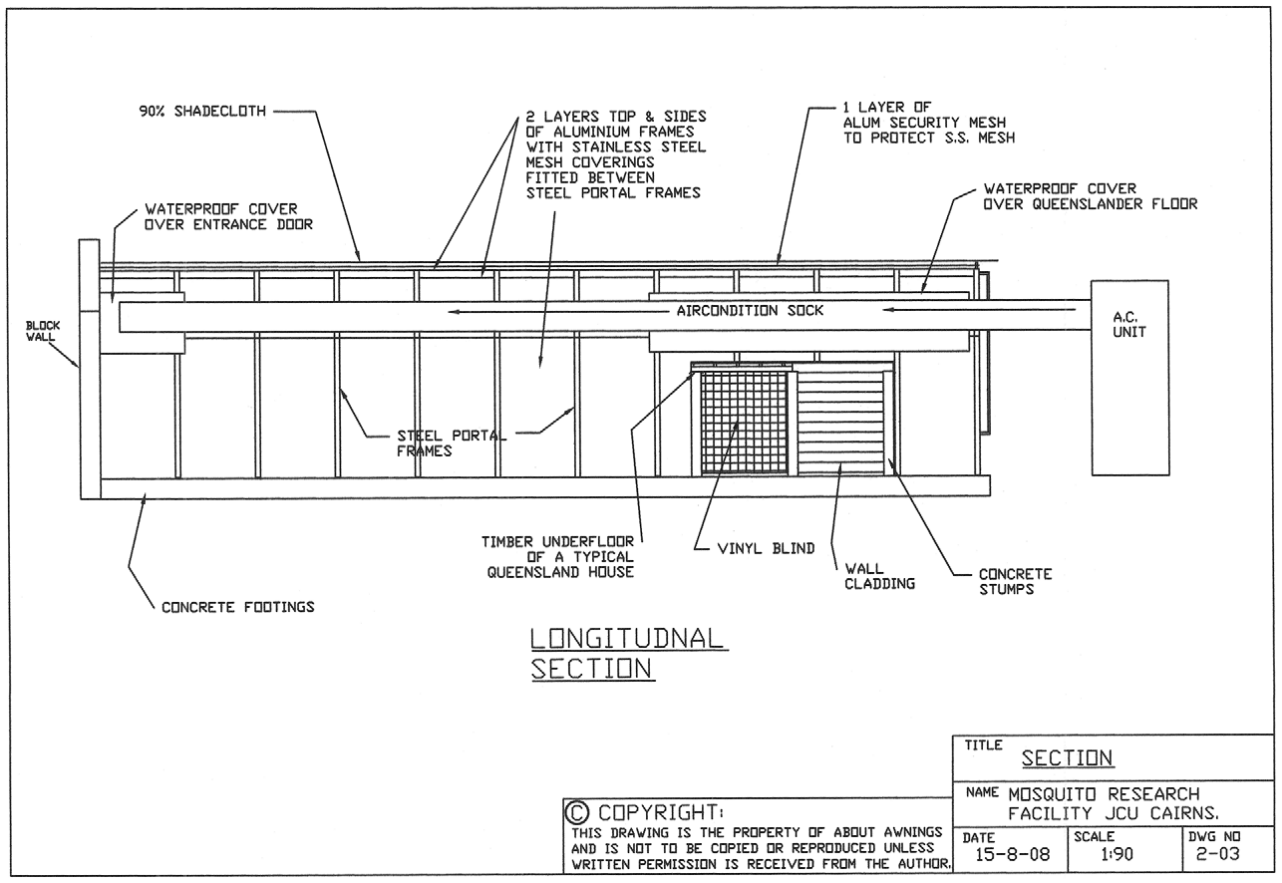
Longitudinal section of James Cook University Mosquito Research Facility semi-field system cage.

**Figure 3:**
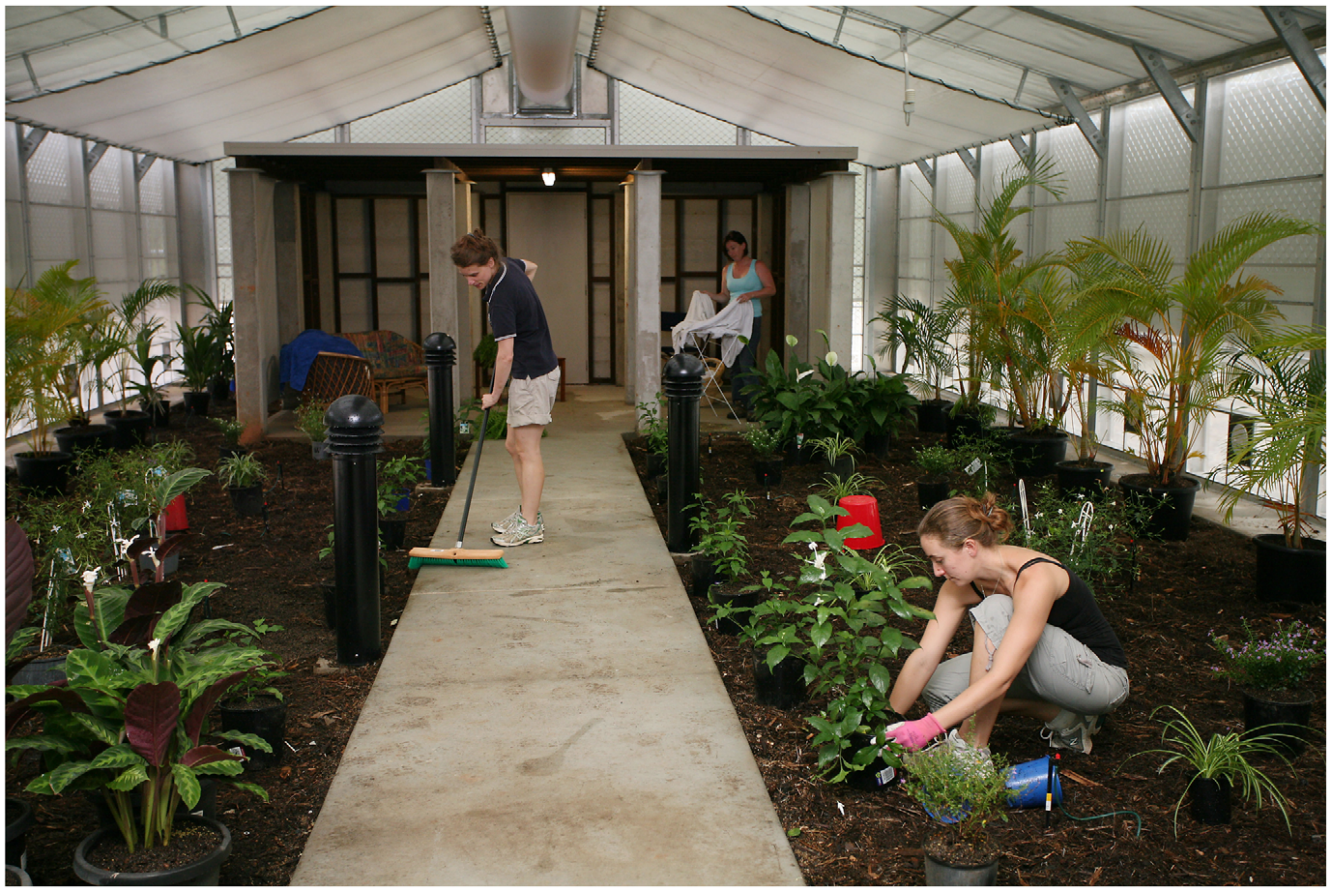
Simulated yard and Queenslander within the MRF SFS. The air distribution sock of the air conditioning system can been seen at the top of the image.

The SFS cages (Figures 1–3) are engineered to the Australian Building Code Cyclone Rating Category 2 to withstand winds up to 216 km/hr (60 m/sec). Each cage is built upon a levelled soil pad with a concrete perimeter, and consists of an inner and outer layer (a “cage within a cage”) separated by 14 cm space. This definitive containment area is required by Australian Quarantine and Inspection Services for identifying possible breaches of the mesh screens. The outer cage measures 18.0 m long×9.0 m wide and 2.8 m and 4.1 m high at the wall and ceiling peak, respectively. The interior cage measures 17.5 m×8.7 m, with a respective height of 2.8 m and 4.1 m at the wall and centre ceiling, for a total interior volume of 465 m^3^. The cage walls consist of 27 1.5 m wide galvanised steel portal frames that support a V-shaped roof consisting of 11 panels connecting to the roof along each side, 5 panels at each rear end, and a solid concrete wall at the front (Figure 2). Each portal frame is individually screened with inner and outer layers of 0.25 mm stainless steel mesh (wire diameter 0.09 mm) separated by 14 cm. Individual portal screens are designed to be removable and repairable in the event of damage. Damage to external screens may not require depopulating of the cage, but severe damage to internal screens that would allow mosquitoes to invade the space between the screens would require cage depopulation. The 27 portal frames sit on a 100 cm×10 cm concrete perimeter slab which is sitting on top of 60 cm deep×20 cm wide concrete rat walls for the entire perimeter of the cages. The cage exterior is covered with 7 cm heavy aluminum security screen over the entire roof and sides to 120 cm off the ground to provide protection from flying debris. The roof and 2/3 of the side walls are also covered with Blue Gum Polyfab 90% shade cloth elevated on galvanised steel pegs and frames 30 cm above the security mesh to minimise solar gain within the cage while maintaining ventilation between the shade cloth and the cage. Waterproof covers are located inside the cages over the Queenslander structure, laboratory entrance, and along the centre of the cage above the air-conditioning sock to protect these areas from rainfall.

The internal residential “Queenslander” structure (4.0×5.0×2.2 m high; internal volume 44 m^3^; Figures 2 and 3) is designed to simulate the ground floor (or the understory) of a Queenslander house based on traditional design and construction materials used in the construction of these houses in north Queensland. Twelve 20×20 cm concrete reinforced posts, set in 4 rows of 3 posts, were sunk 80 cm in to the ground, leaving 210 cm exposed headroom. Each post was topped with a 350 ml diameter galvanised steel pan (ant cap) to prevent access by termites and ants. Wooden bearers (9×7 cm) were laid across three posts at each of the 4 rows. Ten 7×4 cm wooden joists were attached crosswise to the bearer with a galvanised steel cyclone rod. An external grade 19 mm thick plywood ‘floor’ was fixed atop the joists and finished with a weather resistant sealant. Half of the structure has been enclosed with timber stud wall framing and fibro cladding, with a back door fitted to aid in its authenticity. A vinyl blind was added to the front side wall panel to further enclose the structure, leaving on the front aspect of the structure (11 m^2^) open. A fluorescent light tube is attached beneath the joists and power points are located on one of the posts.

The Queenslander structure was fitted with two chairs, a small lounge, a suitcase and small table to create a domestic environment. To further humanise the structure, clothes were hung and used towels, obtained twice/week from a local gymnasium, were scattered on the floor and hung on a clothes horse. A concrete pathway connected the exit door to the simulated Queenslander understory. A residential yard was created outside the house by covering exposed soil in a 10–15 cm deep layer of garden mulch. The mulch was incubated beneath black plastic tarp exposed to sunlight for 24 hours to kill insects within the mulch. Common flowering ornamental plants (*Calyptrocalyx sp., Whitfielda longiflora, Chlorophytum sp., Spathphyllum sp.*, (Peace lily), *Jasminium officinale* (Climbing Jasmin), *Calathea warscewiczii, Euphorbia sp.* (Diamond frost), *Cuphea sp.* and *Dypsis lutescens* (Golden cane)), set in plastic pots, were placed throughout the yard. An automatic sprinkler system with ground-based mister heads was installed under the mulch to water the plants. An attempt was made to kill any arthropods in the mulch by steam-cleaning the mulch after it was laid out. After the initial wet season, the garden mulch, which had become sodden and muddy, was replaced with coir mulch. The coir mulch retained moisture but did not break down as rapidly as the garden mulch.

#### The MRF SFS cages: hydraulics

The MRF drainage system is designed to Australian AQIS containment level QIC2 standards to prevent the egress or ingress of insects. Shallow spoon drains run around the concrete perimeter of the cage and flow into 18 floor drains. Floor drains are screened with a large-particle arrestor trap covered by a fine-mesh filter sock. Each week, the arrestor basket and filter sock are removed and cleaned. Trapped particles are rinsed out and waste particles (mulch and dirt) are either returned to the cage floor or placed in an autoclave bin. Slab wastes drain to the storm water whilst sinks drain to the municipal sewage system. Water can also percolate out through the dirt floor of the cage.

#### The MRF SFS: electrical

The cages are fitted with wall and ceiling mounted fluorescent lights and bollard lights down the walkways. All fittings have been chosen to meet outdoor conditions, and have been installed with sealants to the wiring cavities to prevent the ingress of insects. Several 10 amp power points have been located on the laboratory wall and within the cages. Air curtains located above the vestibule entry doors are programmed to come on 2 seconds prior the door unlocking and only one set of doors can be opened any given time. This can be overridden by disabling the programming system. The vestibule doors are on an alarm system to alert the operators of the facility that doors have been left open. The doors are on self closing operators and the alarm system is provided for back up purposes only.

#### The MRF SFS: climate control

An air conditioning (AC) system was constructed to control temperature and humidity within each cage to match ambient conditions and prevent overheating due to solar gain within the SFS cages. The AC system is comprised of two external AC units that fed cooled, humidified air into each cage. The air is released from the central ceiling beam from a suspended 17 m long, 55 cm diameter polyester cloth air distribution sock (Klimagiel Via XXIV Maggio, 6 Verona Italy; Figures 2 and 3) perforated with 4 mm holes that run the length of the sock. The cloth tube disperses modified air evenly along the cage ceiling while preventing mosquitoes from escaping the cage via the AC duct. Sensors located 2.5 m above ground inside and outside the each cage monitor ambient temperature and relative humidity, with AC controlled to maintain within +/− 1°C and +/− 5% RH of ambient conditions. The air handling units serving the cages constantly ventilate the space during the day. The cooling coil is activated only when a temperature difference of + 0.5°C (internal to external ambient) is detected. The units serving the cages can also be set to artificially increase the RH levels during the dry season if required. Humidity can also be increased by increasing the sprinkler watering time.

### Environmental conditions within the SFS cages

The screening of the cages reduced incoming light and thus potential solar gain. We measured light passing through the cage layers into the SFS cages using a Extech EasyView EA30 light meter (Extech Instruments Corporation, Waltham, MA 02451 U.S.A.) during mid day on clear conditions. We measured temperature and RH inside and outside each cage to test the ability of the shade cloth awning and AC system to maintain ambient conditions. Data loggers (Esis Hygrocon DS1923, Esis Pty Ltd, PO Box 450, Pennant Hills NSW 1715 AUSTRALIA) were set 24 cm above ground on a 8 L plastic bucket located within the Queenslander house and in the yard in the center of each cage, and run while the AC was on and off to investigate the impact of solar gain and the AC unit on conditions within the cages. Outside, 2 data loggers were set, one under a shaded tree ca. 1.5 m off the ground (equivalent to 1.5 m Stevenson screen height used by Bureau of Meteorology) and the other set on a upturned bucket in a shaded area adjacent to cage A. None of the data loggers were exposed to direct sunlight that could heat the unit and provide inaccurate temperature readings.

### Security features of the MRF

Several systems are deployed at the MRF SFS to provide security against vandalism and to minimise the accidental release of insects. The cages are surrounded by 2 m high fencing topped with barbed wire to prevent access by animals and humans. Each cage has an auto-locking door that could only be opened once the entry door into the vestibule was closed. Before being opened, the entry door activated an air curtain above the cage side door that blew air downward over the entryway. The interior also had overlapping screens composed of fine polyester Tentex 72007 cloth (located on the vestibule side) that had a metal chain weight sewn into the bottom to ensure the screens securely overlapped. All doors entering the laboratory are auto-locking, and keys are only available to JCU staff working on the project. The doors have all been fitted with rubber seals. In total, there are 6 doors (3 from cage to insectary, and 3 from insectary to external) between each cage and the external exit of the MRF SFS. Within each vestibule entry into the cage, a BG-Sentinel trap (BGS, Biogents GmbH, Regensburg, Germany) [12] runs continuously and a sweepnet is provided for staff to capture any escaped mosquitoes. All drains within the cages have stainless steel basket screens (0.25 ml) covered with fine mesh socks that are regularly inspected and cleaned. The external and internal walls of the cage are inspected for damage weekly. All supply air and return air grilles are fitted with 0.25 mm stainless steel mesh within the MRF facility. Fire extinguishers are located within the cages and laboratory, and fire detectors are located in the laboratory, air-conditioning system and plant rooms. The building is fitted with a “Notifier” system that automatically dials out to the fire brigade and campus security personnel in the event of a fire alarm. A set of SOPs (File S1) are used to maintain surveillance and security within the MRF SFS.

Extensive monitoring is conducted on the JCU campus to detect mosquitoes that may have escaped the SFS. Sticky ovitraps [13] and 4 BGS traps are also situated in buildings near the MRF, and are serviced weekly. Sticky traps consisting of 700 BGS ml red plastic cups containing a sticky panel insert are placed within the containment space between the mesh layers of each portal frame (Figure S1). If required, breaches of the cage sections can be rectified by replacement of the independently fitted double layers of 0.25 BGS mm stainless steel mesh. Any mosquitoes collected are identified in the laboratory, and *A. aegypti* are sent to University of Queensland for identification of *Wolbachia* infection. The presence of *Wolbachia* was detected by polymerase chain reaction analysis using primers specific to the wMelPop IS5 insertion sequence as described in [14]. Several times a year container surveys are conducted on the JCU campus, and potential *A. aegypti* larval habitat is removed or treated with S-methoprene pellets.

### Distribution of *A. aegypti* within the SFS cages

We sampled cohorts of male and female *A. aegypti* released within the MRF cage to determine their preferred resting sites. Three cohorts of 120 female and 60 male pupae were allowed to emerge in the cages at two day intervals. Mosquitoes were provided with daily human blood meals and access to oviposition sites as per regular experiment procedures. Separate areas in the cages were surveyed with a Prokopack aspirator [15] 3–7 days post-emergence. The cages were divided into five sections; (facing into the cage) left garden, inside Queenslander, right garden, behind Queenslander and front entrance of cage, and were surveyed in that order. Three surveys were performed at around dusk, when mosquitoes were less active, and three surveys were performed in mid-morning prior to blood-feeding. The dusk collections were performed 7, 8, 9 days after the first release of pupae and the day collections were performed 9, 10 and 11 days after the first release of pupae. One person (PHJ) performed all aspirator surveys and followed a specified route around objects (eg, plants, light fittings, furniture, sweaty towels) in each section. Mosquitoes were released back into the cages at the end of each survey. Data for all survey times were combined. For each cage, Fisher’s Exact Test was used to compare the total number of female and male mosquitoes captured within the Queenslander structure compared with those captured elsewhere in the cage.

### Survival of mosquitoes within the SFS cages

A cohort of known numbers of equally aged male and female *Ae. aegypti* were allowed to synchronously emerge in each cage to estimate daily survival within each MRF cage. Mosquitoes (F1 obtained from populations collected from over 280 ovitraps set in suburbs across Cairns) were reared in the MRF insectary as a single large cohort. Larvae were reared in 3.4 L white buckets with approximately 2 L of water (ca. 100–150/bucket) and fed a diet of fish food (Tetramin). Temperature was maintained at 26°C with a 12∶12 photoperiod. Pupae were sexed using size as an indicator and 2500 female and 2500 male 0–24 hr old pupae were placed in buckets and allowed to emerge in each cage (total 5000 mosquitoes per cage).

Mosquitoes within the SFS cages were blood fed on 1–2 human volunteers for 10 min. at around 10 AM each day. Two BGS traps were set in the Queenslanders in each cage and run for 30 min. and the mean number of male and female captured *Ae aegypti* calculated. Samples were not returned to the cages. After 22 days, all remaining mosquitoes were captured using BGS traps and human-bait sweepnet collections.

Mosquito oviposition took place in 8 ovibuckets placed in the yard area of each cage. The ovibucket consisted of a 4 litre plastic bucket filled with 2 BGS L of a 20% hay infusion; a 10×15 cm red flannel cloth strip was attached inside the bucket as an oviposition substrate. Half of the ovibuckets in each cage were changed every three days so each ovibucket was in the cage for 6 days.

Daily survival rates (DSR) were estimated using BGS trap sample and final trap-out data. Both methods of analysis assume that mortality is independent of age and are potentially biased as BGS trap samples were not returned to the cages [16], [17]. However, linear analyses were used for both estimates as the recapture rate was low (overall 10–12% of the total initial population), survival was high and data from the first collection period was very low.

**Figure 4:**
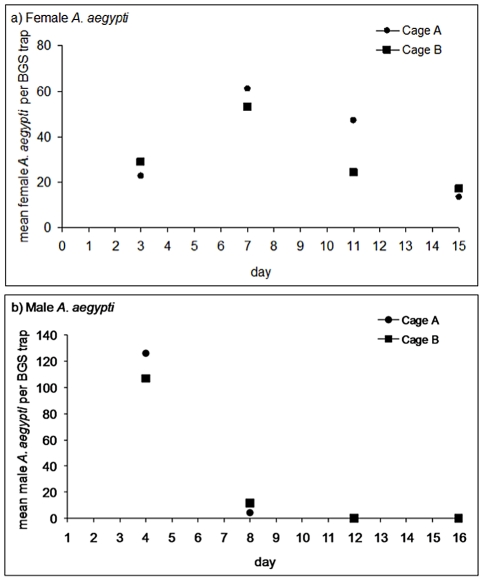
Survival of *A. aegypti* in the MRF SFS. Mean number of female (A) and male (B) *A. aegypti* collected in BGS traps (2 traps/cage) in 30 minutes in each cage.

Mean BGS trap collections (+1) for females and males in each cage were log_e_ transformed and fitted by linear regression against time (day of sample day 0 to day 15 for females and day 11 for males). The DSR were calculated from the resulting slopes [18]. For the DSR estimate for females using log-linear regression, the first sample point on day 3 was excluded from the regression as fewer mosquitoes were collected in the BGS-trap on day 3 than on the next sample day, day 7 (Figure 4A), likely due to a poor collection of teneral adult females by the BGS trap [19] on day 3.

Male DSR were estimated based on samples from day 3 to day 11.

The DSR based on the remaining number of female mosquitoes collected in each cage on day 22 was estimated by solving for p in the exponential decay equation 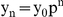
where n is days and y is the number of mosquitoes on that day.

As no males were collected in the final trap-out or in the BGS traps after day 7 (Figure 4B), day 11 and 0 males were used to estimate DSR.

## Results

### Environmental conditions within the SFS cages

**Figure 5:**
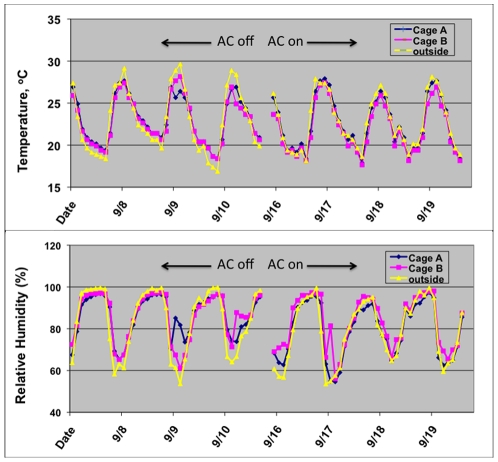
Temperature and relative humidity inside and outside the MRF SFS cages. The air conditioning system was off from 8–10/09/2009 and on from 16–19/09/2009.

Ambient light entering the cage was reduced by 98–99% (Table 1), and was reduced by well over 99% within the Queenslander. Temperature and relative humidity within the cages accurately tracked ambient conditions outside the cage during the Sept 2009 period (Figure 5, Figure S3). Indeed, the AC system appeared to reduce daily peak temperatures by about 2–3°C, suggesting that the shade cloth awning above the cage helped prevent significant solar gain within each cage. Temperature and RH were comparable between the two cages. The mean absolute difference in hourly temperature inside and outside the cage was 0.92 and 1.02°C, respectively, for cage A and B with the AC turned off; and 0.71 and 0.99°C, respectively, with the AC turned on. For RH, the mean absolute difference was 5.6% and 5.5%, respectively, for cage A and B with the AC turned off; and 2.9% and 4.8%, respectively, with the AC turned on. Temperature and RH within the Queenslander were comparable to both outside ambient and yard conditions within each SFS cage (Table 2). The level of solar gain was not high, and reflects the 99% reduction in light entering the cage. Thus, temperature did not become extreme when the AC system was off, although the AC did appear to reduce highest temperatures in the afternoon. Aberrant RH peaks within both cages during the day was caused by water from the automated sprinkler system. Long term temperatures in both cages remained comparable (Figure S3), with cage A ca. 0.5°C warmer than cage B. Especially hot afternoon temperatures in early February 2010 exceeded 35°C, but were nearly identical inside and outside both cages.

**Table 1:** Light in the James Cook University – Mosquito Research Facility semi-field system cage.

Location	Lux	% reduction
Outside cages, full sun	118,000	not applicable
Inside cage, sunlit near entry	2,350	98.01%
Inside cage, under shade of center sock	1,105	99.06%
Inside cage, entrance into Queenslander house	392	99.67%
Inside cage, at back wall of Queenslander house	20	99.98%

Values are mean of peak incidental light measured in lux at ground level using a Extech EasyView EA30 light meter from each cage from 11∶35–11∶55, March 3, 2009.

**Table 2:** Environmental conditions within the MRF SFS cages.

	Cage A		Cage B		Outside
	Yard	Qld	Yard	Qld	
Temp., °C	22.9±3.9	22.5±3.1	22.1±3.7	22.4±3.0	22.8±3.5
RH (%)	79.0±16.4	77.8±12.1	82.4±15.7	80.7±13.2	79.5±14.6

Data are mean daily temperature (°C) and relative humidity (%) readings from data loggers set in simulated yard and Queenslander house (*n* = 2/area) recording every 30 min. from 16–25 Sept. 2009 within cage A and B. Outside readings (*n* = 2) were taken in shade 0.24 m and 1.5 m above ground.

The exterior drainage system prevented overrunning and flooding within the MRF cage due to heavy tropical rains. Indeed, no evidence of flooding within the MRF cage has been observed despite extreme rain events in excess of 300 ml within 24 hr. Overflow of interior drains from rain penetrating the cage screens has also not been observed. The soil base of the cage allows much of the storm water to percolate out of the cage rather than being flushed through the floor drains.

### Biosecurity of the MRF-SFS

There is no evidence of *A. aegypti* escaping from the MRF-SFS. *Aedes aegypti* were occasionally captured in the BGS traps and sticky ovitraps located on the JCU campus. From February to June, 2009 a total of 47 (30 female and 17 male) *A. aegypti* were collected in 4 BGS traps from February to May 2009. During this time 14,800 non-infected and 48,000 *Wolbachia*-infected *A. aegypti* had been released in the cages. But none of the *A. aegypti* collected from the external traps was positive for *Wolbachia* by PCR assay. Whilst the absence of *Wolbachia* does not preclude the possibility that these mosquitoes escaped from the SFS cages, an alternative source of the mosquitoes was usually located. For example, the majority (25/47) of the *A. aegypti* were captured in one fortnight in a BGS trap located near an *A. aegypti* field bioassay from which adult mosquitoes had inadvertently escaped. Also, *A. aegypti* had been detected on campus before the cages were operational. Mosquito trapping and inspections detected larvae in potted plant bases, drain sumps and tyres. Although these sites were treated, breeding may have persisted.

Unwanted arthropods, such as millipedes, phorid flies, ants and some spiders, were observed in the SFS cages. These were probably introduced before screening of the cages was completed, and may also have been entered the cages from contaminated mulch or ornamental plants. Many remained in the cages despite the steam-cleaning of the mulch. Most arthropod populations were self-limiting while spiders and their webs were removed by hand. Ants may been present in the site soil or tunnelled beneath fencing and ratwalls into the cages. These were subsequently controlled by placing ant baits containing AmdroTM (0.73% hydramethylnon) within protective plastic petri dishes inside and outside each cage. A few geckoes (the exotic *Hemidactylus frenatus* (Dumeril and Bibron)) that probably invaded the Queenslander before the cages were screened were also found in each cage. These were removed by hand or by spraying with DettolTM (active ingredient Chloroxylenol (4-chloro-3,5-dimethylphenol)). Whilst the use of Dettol is not an approved method for removing geckos, it was the only effective one available. Spraying Dettol at the gecko would cause it to jump off the wall onto the floor rather than running to a crevice in the Queenslander wall or ceiling. Once on the floor the gecko could be quickly caught and killed by freezing.

### Distribution of *A. aegypti* within the SFS cages

**Figure 6:**
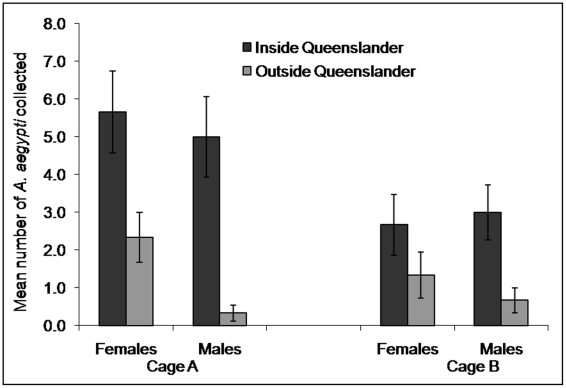
Distribution of resting *A. aegypti* within the MRF-SFS cages. Mean (± SE) number of *A. aegypti* collected with a Prokopack aspirator inside and outside (yard) the Queenslander structure (collection times combined).

For cage A, significantly more *A. aegypti* females and males were collected inside the Queenslander structure compared with all the other areas of the cage (Fisher’s exact test, *p* = 0.02). This was less apparent in Cage B where similar number of females were captured in the Queenslander, (Fisher’s exact test, *p* = 0.32), but fewer mosquitoes were collected overall (Figure 6).

### Estimated daily survival of *A. aegypti* in the SFS cages

The mean numbers of female and male *A. aegypti* collected in the BGS traps was consistent between the two cages (Figure 4; Figure S2). The day 22 trap-out collected 1,073 and 880 females from Cage A and Cage B, respectively; no males were collected from either cage. Estimated daily survival rate of females was similar for both cages across both estimation methods, ranging from 0.92–0.96 (Table 3). The DSRs for males were much lower, but there was nearly a 30% difference between estimates from the two methods, perhaps owing to different termination days.

**Table 3:** Daily survival rate estimates for *A. aegypti* in the MRF-SFS cages.

Method	Females		Males	
	Cage A	Cage B	Cage A	Cage B
log linear regression estimate based on BGS trap data (95% CI)	0.92 (0.87– 0.98)	0.94 (0.92–0.97)	0.77 (0.69–0.85)	0.78 (0.76–0.79)
exponential decay estimate based on final trap-out data	0.96	0.95	0.49	0.49

Mosquitoes collected with BGS traps to 15 days post release. After 22 days, all remaining mosquitoes were captured using BGS traps and human-bait sweepnet collections.

## Discussion

The MRF provides a secure insectary for the production of mosquitoes and replicate quarantine level 2 SFS cages for conducting of experimental releases. The temperature and relative humidity within both SFS cages closely tracks ambient conditions outside the cages. We had fears that solar gain within the cages would result in high daytime temperatures that could be lethal to mosquitoes. Temperatures over 50°C were reported within the SFS in Tanzania, Africa [9]. However, the Tanzanian SFS had no AC system, and no protective awning to reduce solar gain. Our AC system was able to help maintain mean daily maximum temperatures within 1–2°C of external ambient (Figure 5, Figure S3). The multiple layers of shade cloth and screening reduced incident light within the cage by 98–99% (Table 1). This, coupled with ventilation facilitated by the void of 20–30 cm void beneath the shade cloth, ensured that the cage did not heat up appreciably when the AC was turned off (Figure 5). Temperature and relative humidity within the SFS Queenslander were similar to those recorded in the SFS yard, but light incidence was considerably reduced. Temperature and RH with in the SFS Queenslander are comparable to those occurring within a typical well-ventilated Queenslander house. Data loggers set from 1–8 Dec. 2007 in three rooms within a Queenslander house in Cairns demonstrated that average temperature was within 1°C of external Stevenson screen height temperature (S. Ritchie, unpublished data). However, cooler, high-humidity microclimates did exist in sheltered, moist areas such bathroom and laundry. The moist towels placed in the Queenslander within our SFS would have also provided a cooler, high humidity microclimate.

A simple awning system also minimised solar gain and excessive temperature within in two smaller cages (7 m×6 m×4 m high) near the MRF-SFS. These cages were built of 0. 25 ml Tentex polyester covered with a 0.2 m elevated 90% shade cloth awning. The mean maximum daily temperature (from 10 Feb. – 1 Mar 2010 using data loggers set 0.24 above ground) in these cages was only 0.44°C and 0.17°C higher than ambient (J. Darbro, unpublished data). Thus, data from both the MRF-SFS and the adjacent small cages indicate that a simple elevated awning of shade cloth will provide shade and ventilation, preventing high solar gain and extreme temperatures within the cage. This would be a cheaper alternative to air conditioning units.

Aspirator collections within the SFS cages indicated that most mosquitoes harboured within the Queenslander structure. Furthermore, we do not observe large numbers of mosquitoes resting on the cage walls. These observations indicate that the MRF SFS simulates a typical north Queensland urban environment for *A. aegypti*. Daily survival rate of female *A. aegypti* was quite high within the MRF SFS. Estimated daily survival rate of 0.92–0.96 was obtained from a released cohort of females *A aegypti*. Male DSR was much lower, (ca. 0.5–0.78), suggested that they died from starvation due to a lack of food or feeding. Either the flowering plants available in the cage were not suitable, or the males spent less time feeding compared with other behaviours such as mating. However, the high DSR estimates for females may be unrealistic high. Certainly mortality from predation, insecticide exposure and desiccation during prolonged flights are minimised within the cage. Female mosquitoes also had ready and easy access to a blood source (volunteer blood feeders were available every day) and oviposition sites, and thus were likely to expend less energy in searching for hosts or oviposition sites than wild mosquitoes.

We acknowledge that the MRF SFS has limitations. Due to the high construction costs, we were limited to only two SFS cages. Thus, experimental replication will be minimal, requiring multiple sequential experiments in some instances. These experiments could be further complicated by seasonal differences between sequential runs. Environmental conditions within the SFS cage are also different from the natural urban environment. While temperature and RH were comparable to external ambient conditions (Table 2, Figure 5), the screening and shade cloth greatly reduced light and wind within the cage, and the limited space within the cage would may have greatly restricted flight activity. These could impact mosquito survival and the potential infection by agents such as *Wolbachia*. Thus, results from SFS experiments must be interpreted with caution, especially regarding extrapolation to field conditions.

The MRF SFS is a highly secure environment. No *Wolbachia*-infected *A. aegypti* have been detected outside the SFS. Adult mosquitoes would have to escape through a double layer of 0.25 mm stainless steel, limiting these events to a breech of the containment by damage to the structure by flying tree branches, sabotage or vehicular collision. Barring a breech of the cage screening, a mosquito would have to fly through 6 secure locked doors to escape. Both are highly unlikely events. Adult mosquitoes could oviposit in free water or even mulch within the cage. However, all drains have secure 0.25 mm mesh baskets that would contain larvae. The oviposition buckets are the only source of free-standing water in the SFS cage. Thus, larvae hatched from eggs laid on mulch and other wet areas would not develop into adults. Nonetheless, care must be taken to eliminate free standing water in areas like plant axils and drains. In some instances regulatory bodies may require that genetic material not leave the SFS. Water from drainage and direct contact with the soil could allow for transfer of genetic material in our cage without the escape of living mosquito eggs, larvae or adults. A sealed concrete foundation, together with collection of waste water, would have to be used to prevent this. Finally, insects and other animals entered the cage in some instances. Most invaded the cage before it was screened, entered in mulch and plants brought into the cage or may have burrowed from the soil. Care must be taken to ensure contamination is minimal, and harmful mosquito predators, such as ants and geckoes, are eliminated.

Nonetheless, contained SFS cages offer excellent opportunity to conduct research on insects. The secure environment prevents release of quarantine insects; to date, no *Wolbachia*-infected *A. aegypti* have been detected in surveillance traps on the JCU campus. The cage allows for the release of cohorts of known numbers. Thus, the direct impact of a control measure can be estimated by comparing changes in population between control and treatment cages. This approach as been used to study the impact of pesticides, repellents and parasite-vector interactions (for a review see Ferguson et al. [9]). Cohort cage studies can also be used to study the behaviour of mosquitoes [9], and to estimate the relative efficacy of traps [20]. The MRF SFS could also be used to conduct insecticide and repellent evaluations under controlled semi-field conditions without the ethical dilemma of disease risk. We hope to investigate the impact of competing oviposition containers on efficacy of ovitraps such as sticky ovitraps and lethal ovitraps. Furthermore, detailed studies on *A. aegypti* behaviour, such as the microclimate of preferred harbourage sites, can be conducted on released cohorts within the Queenslander structure. While we have not established populations within the cage, we believe it would be relatively easy to do so as has been done with *Anopheles* [9].

For our studies with *Wolbachia*, we will be able to observe the rate of *Wolbachia* invasion within a population of wild *A. aegypti*. These studies will measure the penetration of *Wolbachia* within wild *A. aegypti* after simultaneous release of known ratios of *Wolbachia*-infected and uninfected *A. aegypti*. This will occur over several generations and be used to estimate the time to fixation We are currently conducting invasion experiments using the *w*MelPop and *w*Mel strains.

## Supporting Information

Figure S1 **Escape of mosquitoes from interior of SFS cage is monitored by sticky trap set in space between the two stainless steel layers of MRF-SFS cage.**
(3.83 MB TIF)

Figure S2 ***Aedes aegypti*** **daily survival rate estimate based on mean recaptures in BGS traps (loge+1 transformed).** a: Cage A Female *A. aegypti*; b: Cage B Female *A. aegypti*; c: Cage A Male *A. aegypti*; d: Cage B Male *A. aegypti.*
(0.19 MB TIF)

Figure S3 **Temperature in the MRF SFS cages tracks ambient external temperature.** Values are mean daily minimum and maximum temperature within MRF cage A and B, and Bureau of Meteorology data collected 10 km from the site (from 25 Sept 2009 – 9 Feb. 2010).
(0.37 MB TIF)

File S1 **Standard operating procedures for the James Cook University Mosquito Research Facility semi-field system.** Updated February 2009.
(0.14 MB DOC)
