# Supplementary figures and images for: A Secure Semi-Field System for the Study of Aedes aegypti
Source: PLoS Negl Trop Dis. 2011 Mar 22;5(3):e988. doi: 10.1371/journal.pntd.0000988 (PMC3062535; doi:10.1371/journal.pntd.0000988)

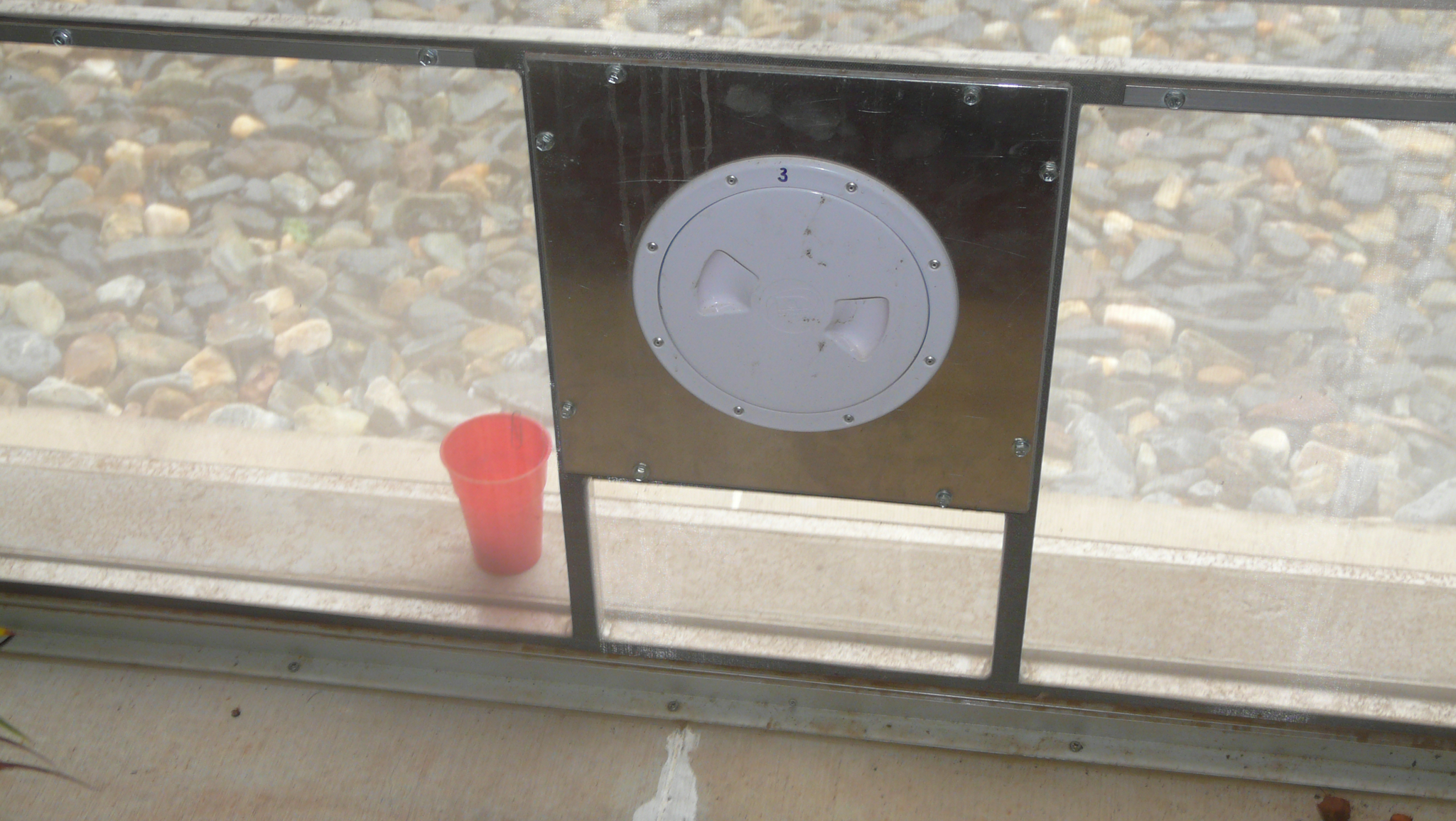

Supplement: Figure S1 — Escape of mosquitoes from interior of SFS cage is monitored by sticky trap set in space between the two stainless steel layers of MRF-SFS cage. (3.83 MB TIF) [file pntd.0000988.s001.tif]

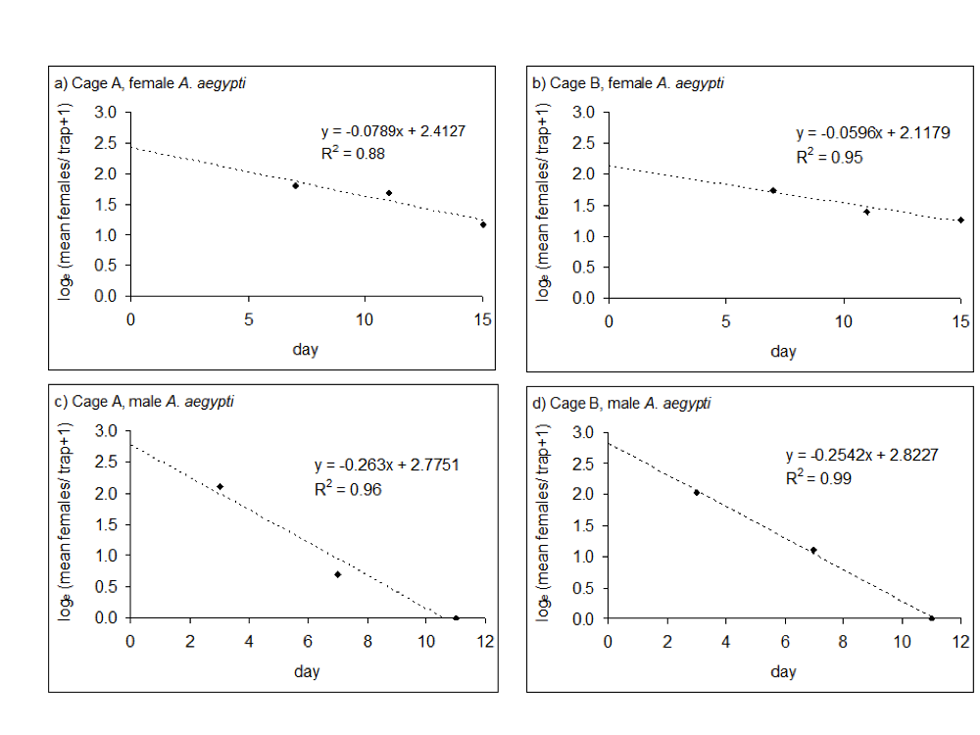

Supplement: Figure S2 — Aedes aegypti daily survival rate estimate based on mean recaptures in BGS traps (loge+1 transformed). a: Cage A Female A. aegypti; b: Cage B Female A. aegypti; c: Cage A Male A. aegypti; d: Cage B Male A. aegypti. (0.19 MB TIF) [file pntd.0000988.s002.tif]

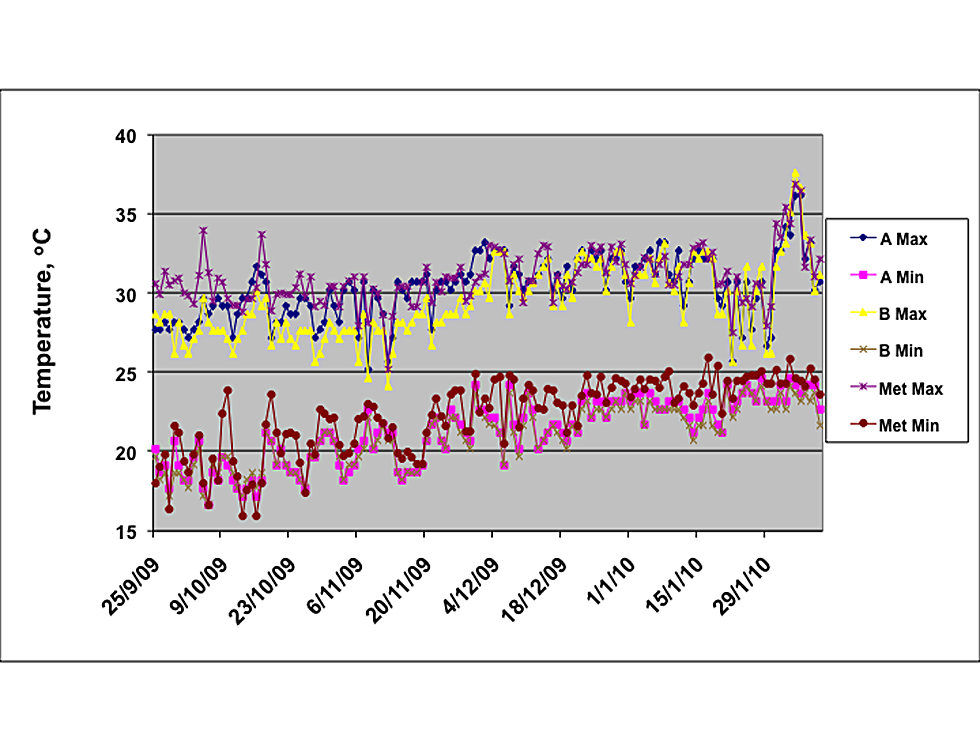

Supplement: Figure S3 — Temperature in the MRF SFS cages tracks ambient external temperature. Values are mean daily minimum and maximum temperature within MRF cage A and B, and Bureau of Meteorology data collected 10 km from the site (from 25 Sept 2009 – 9 Feb. 2010). (0.37 MB TIF) [file pntd.0000988.s003.tif]
