# Supplementary material for: A Secure Semi-Field System for the Study of Aedes aegypti
Source: PLoS Negl Trop Dis. 2011 Mar 22;5(3):e988. doi: 10.1371/journal.pntd.0000988 (PMC3062535; doi:10.1371/journal.pntd.0000988)
Supplement: File S1 — Standard operating procedures for the James Cook University Mosquito Research Facility semi-field system. Updated February 2009. (0.14 MB DOC) [file pntd.0000988.s004.doc]

**File S1. Standard operating procedures for the James Cook University Mosquito Research Facility**

**semi-field system.** Developed and updated February 2009.

[1.1 Emergency Responses 2](#__RefHeading___Toc226342927)

[1.1.1 Minor breach 2](#__RefHeading___Toc226342928)

[1.1.2 Major breach 2](#__RefHeading___Toc226342929)

[1.1.3 Rapid depopulation of cages in response to an emergency 2](#__RefHeading___Toc226342930)

[1.1.4 Follow-up procedures in response to a major breach and/or emergency depopulation of cages 2](#__RefHeading___Toc226342931)

[1.2 Other emergency response situations 3](#__RefHeading___Toc226342932)

[1.2.1 Cyclone (severe weather) 3](#__RefHeading___Toc226342933)

[1.2.2 Flood 3](#__RefHeading___Toc226342934)

[1.3 Containment and surveillance 5](#__RefHeading___Toc226342935)

[1.3.1 Entry and Exit of Facility 5](#__RefHeading___Toc226342936)

[1.3.2 Surveillance of laboratory building (includes CT rooms and tea room) 5](#__RefHeading___Toc226342937)

[1.3.3 Monitoring and surveillance of cages 6](#__RefHeading___Toc226342938)

[1.3.4 Surveillance of environment immediately surrounding the facility 6](#__RefHeading___Toc226342939)

[1.3.5 Surveillance of mosquito populations at JCU Cairns campus 7](#__RefHeading___Toc226342940)

[1.4 Maintenance - cage 7](#__RefHeading___Toc226342941)

[1.4.1 Cage air-conditioning system monitoring and maintenance 7](#__RefHeading___Toc226342942)

[1.4.2 Inspection for predators 8](#__RefHeading___Toc226342943)

[1.4.3 Internal drains 8](#__RefHeading___Toc226342944)

[1.4.4 Garden maintenance 8](#__RefHeading___Toc226342945)

[1.4.5 Cage exterior 9](#__RefHeading___Toc226342946)

[1.4.6 Humanising cage 9](#__RefHeading___Toc226342947)

[1.5 Maintenance – laboratory 10](#__RefHeading___Toc226342948)

[1.5.1 Cleaning schedule 10](#__RefHeading___Toc226342949)

[1.5.2 Waste treatment and disposal 10](#__RefHeading___Toc226342950)

[1.5.3 Laboratory drains 11](#__RefHeading___Toc226342951)

[1.6 Scientific procedures: 12](#__RefHeading___Toc226342952)

[1.6.1 Blood feeding mosquitoes in flight cages and laboratory 12](#__RefHeading___Toc226342953)

[1.6.2 Rearing and maintaining colonies 12](#__RefHeading___Toc226342954)

## Emergency Responses

### Minor breach

- A minor breach – damage to one of the mesh layers
  1. If safe to do so, attempt to prevent further damage or escape of mosquitoes (eg plug holes, repair damage with patch).
  2. Inform supervisor who will arrange for permanent repair.
  3. If the problem becomes worse and potentially could result in a major breach, refer immediately to procedures for a major breach in Section 1.1.2.
  4. Register details of response in log book.

### Major breach

- A major breach – damage to both mesh layers that could result in immediate release of mosquitoes into the environment
  1. If safe to do so, attempt to prevent further damage or escape of mosquitoes (eg plug holes, repair damage with patch).
  2. Inform supervisor to confirm that experiments will be terminated and the cages depopulated.
  3. The decision-making hierarchy is: 1. Scott Ritchie, 2. Scott O’Neill and 3. Petrina Johnson. Contact details are located next to the phones in the analytical area and the tea room.
  4. Carry out depopulation measures (Section 1.1.3)
  5. Register details of response in log book.

### Rapid depopulation of cages in response to an emergency

- 1. Aerosol cans of non-residual, fast knockdown synthetic pyrethroid (Mortein Naturgard Fly Spray) will be used to rapidly kill free-flying mosquitoes in the flight cages.
  2. Begin spraying the interior of the Queenslander, and then any other potential mosquito harbourage areas within the cages. Hand nets may also be used to capture and kill mosquitoes.
  3. A depopulation ‘kit’ for emergency procedures is in the marked boxes in the connecting spline.
  4. After the cages are depopulated, continue response with follow-up procedures in Section 1.1.4.

### Follow-up procedures in response to a major breach and/or emergency depopulation of cages

- 1. If safe to do so, continue to inspect cages for mosquitoes by conducting landing catches and running BGS traps in cages.
  2. Increase surveillance trapping around the facility and the campus. Deploy additional sticky oviposition traps (double oviposition traps with sticky panels) and BGS traps.
  3. Any *Ae. aegypti* captured should be preserved in 80% EtOH and tested for *Wolbachia* infection. Note whether the mosquito was freshly killed when preserved. (NB: Not all *Ae. aegypti* in the cage will be infected with *Wolbachia.* Therefore, a negative result does not wholly discount the possibility that mosquitoes have escaped from the facility.)
  4. Notify JCU Facilities Maintenance Staff and Security of the situation. Contact details are located next to the phones in the analytical area and the tea room.
  5. Engage the Queensland Health Dengue Action Response Team (DART) to conduct mosquito control measures on campus (e.g. locate and treat actual and potential breeding sites).
  6. Register details of response in log book.

## Other emergency response situations

### Cyclone (severe weather)

- 1. If a cyclone is imminent, official cyclone warnings will be monitored to assess the level of threat to the site and to determine if experiments will be terminated and the cages depopulated.
  2. The cages will be depopulated if there is threat of a cyclone of category 4 or greater that is predicted to pass within 50km of the facility and/or will result in predicted wind speeds of 160km or greater for the area.
  3. The decision to depopulate the cage will be made 24 hours in advance of predicted landfall.
  4. The Bureau of Meteorology website and weather warning postings will be monitored to assess the threat [http://www.bom.gov.au/weather/cyclone/].
  5. The decision-making hierarchy is: 1. Scott Ritchie, 2. Scott O’Neill and 3. Petrina Johnson. Contact details are located next to the phones in the analytical area and the tea room.
  6. Staff may be required to be on standby to assist with depopulation of cages if necessary. Contact details are located by the phones.
  7. Refer to Section 1.1.3 for rapid depopulation measures, if the decision is made to depopulate cages.
  8. After depopulating cages, remove any moveable, loose objects from cage and store in the Queenslanders or connecting spline. Large objects, especially in the Queenslander structure may be secured with bungy cords. Small plants may be stored in the Queenslander and large palms should be moved away from the cage mesh and laid on their sides.

### Flood

- 1. The risk of severe flooding in the cage from heavy/prolonged rainfall, or from a storm surge is minimal.
  2. If a flood is predicted to occur and sufficient warning has been given, the cage will be depopulated.
  3. The decision-making hierarchy is: 1. Scott Ritchie, 2. Scott O’Neill and 3. Petrina Johnson. Contact details are located next to the phones in the analytical area and the tea room.
  4. Staff may be required to be on standby to assist with depopulation of cages if necessary. Contact details are located by the phones.
  5. Refer to Section 1.1.3 for depopulation measures, if the decision is made to depopulate cages.
  6. After depopulating cages, remove any moveable, loose objects from cage and store in the Queenslanders or connecting spline. Large objects, especially in the Queenslander structure may be secured with bungy cords. Small plants may be stored in the Queenslander and large palms should be moved away from the cage mesh and laid on their sides.

## Containment and surveillance

### Entry and Exit of Facility

- 1. Lab coats must be worn at all times in the laboratory and rearing rooms, but are to be removed before entering cage. Lab coats are located in the main entry antechamber.
  2. Before entering the cages, the lab coat may be removed and placed on coat hook in connecting spline.
  3. Enter and exit from the cages via the connecting spline only. The doors in the antechamber that open to the outside are only to be opened in an emergency evacuation situation.
  4. When entering cages, pass through the overlapping curtains, allowing the fabric to brush against the body. The curtain should not be held open.
  5. Extreme care must be taken when moving equipment around in the cage to prevent damage to the stainless steel mesh. For example, use two people to move large objects around, with a person holding on to both ends of the object.
  6. Do not rest any equipment on the mesh.
  7. Ensure palm fronds do not pierce the mesh.
  8. Refer to Section 1.1.1 or 1.1.2 if cage mesh is damaged.
  9. Before exiting cages, first stand down the Queenslander end of the cage, ensure no mosquitoes are bloodfeeding, then move quickly to the exit.
  10. Press exit button and stand away from the door.
  11. When the door lock has been deactivated, stand and pause beneath the overhead fans and open the door and step in behind the curtain.
  12. Close the door and pass through the curtains allowing the fabric to brush against the body. The door curtain should not be held open.
  13. Do a visual inspection of the anteroom for loose mosquitoes before exiting the anteroom into the connecting spline. Take care to inspect the walls for resting mosquitoes and around feet for flying mosquitoes.
  14. If a mosquito has escaped from the cage into the anteroom, it must be caught (using spray bottle with 80% EtOH, or hand net) before leaving. Confirm identification and sex and record details in log book provided in anteroom.
  15. Lab coat must be worn again before entering the lab.
  16. When exiting the main lab, inspect clothing in the anteroom to ensure no mosquitoes are present. Any mosquitoes present in the anteroom must be immediately killed. Remove lab coat before exiting to tea room.

### Surveillance of laboratory building (includes CT rooms and tea room)

- BGS traps are run in several locations within the facility to capture any free-flying mosquitoes and monitor for containment breach.
  1. Inspect BGS traps once per week.
  2. Ensure trap is working properly (repair or replace trap if necessary) and empty contents of BGS trap bag (change bags.)
  3. Nine BGS traps are located in the facility - Cage A antechamber, Cage B antechamber, connecting spline, CT Room 1, CT Room 2, Animal Room, laboratory, entry antechamber, tea room. These traps are run continuously.
  4. Identify contents of BGS bags.
  5. Complete log book.
  6. If *Ae. aegypti* are found in the traps, notify supervisor.
  7. A staff meeting may need to be called and operating procedures reviewed if *Ae. aegypti* are regularly being collected in traps.
  8. Internal drains are inspected every Monday (Refer to Section 1.5.3).

### Monitoring and surveillance of cages

- The flight cages will be monitored for potential and actual containment breach
  1. Every Monday morning cages are visually inspected first externally and then internally. A complete inspection should also be performed prior to the commencement of each experiment.
  2. Inspection should examine for physical breaches in cages (holes in mesh), obvious presence of insects between mesh layers.
  3. After structural inspection, the gaps between the mesh layers are checked. Visually inspect the sticky resting traps and the floor of each section for dead insects. All 27 sections per cage are inspected.
  4. If any breaches are detected (physical damage or presence of insects between mesh layers) then immediately report to the supervisor who will assess the breach and advise action (Section 1.1).
  5. Insects found in between the layers need to be removed from the floor/sticky trap and identified in the main lab. Two people are needed to inspect the trap, one person to open and retrieve the trap and a second person to fan air over the portal to prevent ingress of mosquitoes from the cage into the gap via the open portal.
  6. Log book completed for items 2, 3 and 6 after completion.
  7. Every four weeks sticky resting traps are replaced. Register in log book when traps are changed. Two people are needed to inspect the trap as described in item 5.
  8. Internal drains are inspected every Monday (refer to Section 1.4.3).

### Surveillance of environment immediately surrounding the facility

- Oviposition traps will be placed in vegetated areas immediately surrounding the facility to monitor for containment breach.
  1. Every week, sticky oviposition traps are inspected.
  2. Four sticky oviposition traps (double sticky ovitraps) are located in the sheltered areas surrounding the facility.
  3. Each trap is examined for presence of adult mosquitoes on the sticky panel on the upper section of the trap. Remove adult mosquitoes from sticky panel using a toothpick, and place in vial for identification.
  4. Every second week change water in the trap and add 1 lucerne pellet and 1 methoprene pellet.
  5. Change sticky ovitrap sticky panel every 4 weeks, or when the panel is very dirty (e.g. after the grass has been mowed or a gecko is stuck to it).
  6. Identify mosquitoes in main lab.
  7. Complete log book for items 4-6.
  8. If *Ae. aegypti* are found in any of the double ovitraps, then immediately notify the supervisor who will assess the breach and advise follow up procedures (Section 1.1).
  9. Preserve *Ae. aegypti* in 80% EtOH, in labelled 5ml vial for testing for *Wolbachia* infection.

### Surveillance of mosquito populations at JCU Cairns campus

- Mosquito traps will be placed around the JCU campus to monitor local mosquito populations and to monitor for containment breach.
  1. Every week, inspect campus BGS traps.
  2. Ensure traps are working properly (repair or replace trap if necessary) and empty contents of BGS trap bag (change bags).
  3. Five BGS traps are located around the campus - Building E1 (downstairs), Building E2 (downstairs), Jellyfish Compound, library and refectory area.
  4. Identify contents of BGS bags.
  5. Complete log book for items 3 and 4.
  6. If *Ae. aegypti* are found in any of the BGS traps, then immediately report to the supervisor.
  7. An inspection of cages for evidence of a breach should be conducted (Section 1.3.3).
  8. Also consider the possibility that *Ae. aegypti* in campus monitoring traps may be originating from an external source.
  9. Follow-up procedures should also be conducted (Section 1.1.4) that will include an inspection carried out by the Queensland Health Dengue Action Response Team to identify and treat any *Ae. aegypti* breeding sites on campus.

## Maintenance - cage

- Containment procedures detailed in Section 1.3.1 must be followed at all times when working in cages.
- Standard PC2 laboratory procedures must be adhered to at all times in the laboratory. These are listed in the entry antechamber and in the main lab.
- Note that insecticide and personal repellents are not permitted in the cages.

### Cage air-conditioning system monitoring and maintenance

- Temperature and humidity monitoring
  1. Temperature and humidity will be monitored through i) daily reading of maximum and minimum conditions from digital weather station, ii) a fortnightly inspection of conditions from ibutton data loggers and iii) a fortnightly inspection of conditions recorded by the air-conditioning system (accessed through JCU Facilities and Maintenance Office).
  2. Every morning, check maximum and minimum temperature and humidity levels recorded by the digital weather station and record in the log book.
  3. If conditions are abnormal (very high or very low temperature, and/or very low humidity) notify supervisor immediately.
  4. Reset the data logger.
  5. Every second Friday morning, download data from ibuttons located in each cage and under trees behind the Marine Research Compound.
  6. In addition, confirm that the weekly record of cage temperature and humidity levels has been sent via email from JCU Facilities and Maintenance Office.
  7. Compare ibutton data with air-conditioning system data. Report any anomalies to supervisor who will notify JCU Facilities and Maintenance Office and/or air conditioner contractors. Contact details are located by the phones in the analytical area and tea room
  8. Register check in log book.
- Air filter maintenance
  1. External and internal air filters to the cages and labs will be maintained by the JCU Facilities and Maintenance and the air conditioner contractors. Lab filters (internal and external) will be serviced every month and cage filters (external filters and barrier screens, there are no internal filters in the cages) will be serviced every four months.
  2. Completion of filter servicing is recorded in the log book. If a service has not been conducted by the expected date, then notify JCU Facilities and Maintenance Office.
  3. After a service, check temperature and humidity conditions in cages and lab to ensure the air-conditioning systems are working properly.

### Inspection for predators

- The cage environment, including the Queenslander structure, needs to be regularly inspected for predators that might have entered cages with plants or potentially through a cage breach.
  1. Every Monday morning, inspect cage for presence or evidence of predators such as spiders (webs or jumping spiders) and geckos (look for faeces). This can be done whilst inspecting for containment breach.
  2. Catch and kill predators to ensure predator populations in the cage are minimal.
  3. If predators are found undertake an inspection of cage according to Section 1.3.3 to ensure that the cage has not been breached.

### Internal drains

- 1. Check internal drains every Monday.
  2. Remove all debris (mulch, leaves) from the around the drain opening and from within the drain filter basket.
  3. Organic debris can be returned to the ‘garden’ within the cages.
  4. Remove the drain filter sock and replace old filter sock with a new/clean one.
  5. Discard old sock if it has holes or is torn. For intact socks, dispose of any solid waste in Autoclave Waste Bin. Soak socks in bleach (20% solution) for at least 4 hours then clean thoroughly in sink. Clean and intact socks can be reused.

### Garden maintenance

- 1. Pot plants and garden mulch are watered between 2:00 and 4:00am (one hour in each cage) every second day (may vary according to season). A timed watering system has been set up in the cage for this purpose.
  2. Proper functioning of watering system is checked in the morning after watering by running the system briefly and ensuring that all pot plants are being watered.
  3. Hand water plants that are not reached by the automatic system (eg hanging ferns).
  4. Check cages daily for fallen palm fronds. Remove fronds and place in ‘General Waste’ Bin (will need to be cut up).
  5. Prune any live palm fronds that are close to the mesh.
  6. Plants will require feeding with slow release fertiliser every month.
  7. Record checks and maintenance details in log book.

### Cage exterior

- 1. Every first Friday Monday of the month, treat periphery of facility with ant sand.
  2. If additional ant control is needed, contact JCU Facilities and Maintenance Office, to arrange for a licensed pest inspector to provide alternative treatments. A supervisor must be present during the inspections.
  3. At the same time, remove weeds from gravel area around cages and ensure that grassed area near lab has been mowed.
  4. Record checks and maintenance details in log book.

### Humanising cage

- 1. Used gym towels are used for humanising the cage and remain in the cages for one week. Two batches of towels per cage are rotated, one batch on Tuesday morning and one batch on Friday morning.
  2. Every Tuesday and Friday mornings, towels are collected from the gym (contact details are located next to the phones and on the notice board) and old towels are returned on the same day.
  3. When removing towels from the cages ensure that mosquitoes are not trapped amongst the towels or escape from the cage as the towels are removed.
  4. Whilst standing in the Queenslander, shake 2-3 towels to remove mosquitoes then place the towels in a laundry basket placed away from the Queenslander. Repeat this until all towels are in the basket.
  5. Move the basket into the antechamber and ensure that no mosquitoes are on or amongst the towels. The towels can be transferred to the gym laundry bag in the antechamber or the spline.

## Maintenance – laboratory

- Containment procedures detailed in Section 1.3.1 must be followed at all times when working in cages.
- Standard PC2 laboratory procedures must be adhered to at all times in the laboratory. These are listed in the entry antechamber and in the main lab.
- Note that insecticide and repellents are not permitted in the cages.

### Cleaning schedule

- 1. Empty rubbish bins according to procedures in Section 1.5.2.
  2. Every Friday afternoon at the end of the day sweep and mop floors in all areas apart from the tea room and toilet.

### Waste treatment and disposal

- Solid waste
  1. Solid waste is separated into General Waste bins or Autoclave Waste bins.
  2. All biological material (eggs, rearing colony material) must be autoclaved before disposal. Note that as long as fallen palm frond are removed from the cages every day or every second day, they can be placed in General Waste bins.
  3. Autoclave Waste must go into designated autoclave bags. Autoclaving is to be done every Friday.
  4. Partially seal bag leaving a fist sized opening at the top so steam can enter and penetrate bag and then temporarily seal the opening with rubber bands for transport to the autoclave.
  5. Place the bag in clearly labelled 500L container with secure lid to transfer the waste to the autoclave machine located in Building E1, Room 103N. Autoclaving should be carried out immediately following waste removal from the facility.
  6. Sterilisation will be conducted at 121^o^C for 30 minutes at 20 p.s.i. (ensure bag is partially open to allow steam penetration).
  7. Log details of autoclave process.
  8. After sterilisation, dispose of bag in the biological waste bin located in the loading bay of building E1.
  9. Dispose of General Waste in the same bin every Friday.
- Liquid waste
  1. No live material will be washed down the sink.
  2. Any liquid waste that may contain live material (eggs, larvae pupae from rearing cultures) is treated by freezing.
  3. Empty all rearing culture water into designated stainless steel buckets and freeze for 24 hours.
  4. Sugar/honey sponges from colonies may have eggs on them and should be treated with bleach (20% solution) before rinsing in the sink.
  5. Treated (frozen) liquid waste will be disposed of outside the facility or within the cages to avoid blocking the sinks.
  6. If large quantities of liquid waste have been produced (e.g. during mass rearing situations), then material may be frozen directly in rearing buckets for 24 hours and then disposed of in the garden area directly outside the facility or in the cages.

### Laboratory drains

- 1. Clean sink filter and socks every Monday.
  2. Remove the drain filter sock and replace old filter sock with a new/clean one.
  3. Discard old sock if it has holes or is torn. For intact socks, dispose of any solid waste in Autoclave Waste Bin. Soak socks in bleach (20% solution) for at least 4 hours then clean thoroughly in sink. Clean and intact socks can be reused.

## Scientific procedures:

### Blood feeding mosquitoes in flight cages and laboratory

- 1. All staff who volunteer for blood feeding must read and complete the Informed Consent for Human Blood-feeding of Laboratory Mosquitoes form in the presence of a supervisor.
  2. Immediately prior to blood feeding, ear temperature must be taken and recorded in log book.
  3. If the volunteer’s temperature is above 38°C, then they will be excluded from feeding.
  4. Mosquitoes will be allowed access to blood feed for 10 – 20 minutes.
  5. Analgesic cream, an ice pack or hot water may be applied after feeding to reduce discomfort and inflammation.

### Rearing and maintaining colonies

- These rearing procedures are modified from laboratory rearing procedures used in the O’Neill lab at University of Queensland.

#### Colony stock

- 1. All stock lines should be screened for infection with *Wolbachia* at least once every 2-3 generations, to monitor infection levels in each line.
  2. Collect samples of mosquitoes in 80% EtOH and send to the University of Queensland (O’Niell lab) for analysis.
  3. When screening for infection the Squash buffer technique for DNA extraction can be used for early instar larvae and adult males using IS5 Primers, while the DNEasy kit will work with all stages except pupae (pupae do not work well when attempting to extract DNA).
  4. All lines are maintained in constant temperature controlled insectaries at 26±1°C, 80-90% RH and with a photoperiod of 12 hr L:D.
- Note that wMelPop *Ae. aegypti* require special attention in two areas: i) blood-feeding (should only be maintained on human blood), and ii) conditioning and storage of egg material (eggs should not be stored for long periods of time between generations).

#### Adults

- 1. Adults are allowed to emerge into adult cages (stocked at a density approximately 600 (approximately 300 females and 300 males per cage).
  2. The cage must be clearly labelled with colony type, generation and date the pupae were introduced to the cage.
  3. The cage is covered with black plastic to maintain high humidity levels. These should be changed weekly.
  4. Honey/sugar solutions are provided to all mosquitoes on the day the emergence cups are placed in the cages. Strips of clean pink sponges are placed in the lid of small sample cups filled with water and the top of the sponge dipped in 10% honey solution. These are changed on Mondays, Wednesdays and Fridays. Used cups are cleaned in a weak bleach solution and rinsed thoroughly. Raisins, sugar cubes and fruit slices may also be used as a carbohydrate source, these must be changed every 2 days.
  5. After emergence females and males require a period of 2 days to complete development (a sugar source must be available during this time).
  6. Colony material is usually allowed to mate for 3 days prior to blood-feeding.
  7. Females become capable of blood-feeding 2-4 days after emergence. Mosquitoes may be fed daily or on Mondays, Wednesdays and Fridays. It is important not to delay bloodfeeding in order to reduce amount of stress on infected mosquitoes and so place pressure on the *Wolbachia*.
  8. Mosquitoes are allowed to feed for 10-15 minutes on the limb of a volunteer. If the majority of female mosquitoes have become completely engorged there will be no need to bloodfeed again before eggs are collected, however if not all individuals have fed well it is a good idea to do a ‘catch-up’ feed (about 5 minutes) the following day in order to ensure a good amount of eggs will be laid during oviposition.
  9. After blood-feeding the eggs take 3 days to develop before oviposition can occur.

#### Eggs

- Oviposition
  1. Three days after a human blood-meal 1 or 2 oviposition cups are introduced into the cage, if this occurs after 96hours and mosquitoes are left without a laying substrate they will become stressed so it is important to remember this step.
  2. Oviposition cups consist of a cup half-filled with distilled water and lined with sandpaper as an oviposition substrate. Oviposition cups should be left in the cage for 2 days to allow all mosquitoes to lay. After 2 days, the sandpaper is removed from the cups and placed on a strip of damp (not sopping wet) paper towel in a sealed plastic container to allow for egg embryonation and conditioning. If the paper towel is too wet, the eggs may hatch precociously and if the paper towel is too dry the eggs will not embryonate.
  3. To properly condition eggs for storage, all dead mosquitoes should be removed from the egg papers and water, this will help to prevent fungal growth occurring later.
  4. After 3 days, the lid is removed from the container and the and the sandpaper allowed to dry slowly.
  5. Eggs are then transferred to a larger egg storage container that has a humidifying solution (salt). Eggs should be checked every fortnight for mould growth.
  6. Note that wMelPop should be hatched 5-8 days after oviposition. Hatching the eggs from these lines within a 3-day window from 5 days post collection gives a hatch rate around 80%-90%.
- Egg storage
  1. For long-term storage of eggs, place the sandpaper containing eggs in a sealed plastic container with a saturated solution of potassium chlorate and store at 15°C. (KClO3 maintains the humidity at an optimum 85%.) The eggs of uninfected *Ae. aegypti* strains have a half-life of up to a year if maintained under these conditions.
  2. Alternatively, eggs may be transferred to a large plastic egg storage container with a salt solution and stored at the airconditioned lab temperature of approximately 22°C
  3. Extra care must be taken when drying down eggs for hatching after periods longer than 1month. All fragments of mosquitoes must be removed from paper before ‘conditioning’ and any paper with blood stains on it should be trimmed to prevent the growth of mould which will inhibit hatching.

#### Larval rearing protocol

NB CT room temperature is 26°C.

- Egg hatching

1. Day 0. Prepare hatching solution with 0.25g bakers yeast added to 1L water (a ‘yeast solution bucket’ and a special measuring spoon is available in each CT room), ensure solution is mixed and use immediately. Hatching solution should not be prepared >10 minutes in prior to use. Use to a depth of approximately 12 cm in rearing container, and add egg papers/ovistrips.

Rearing containers are 3-4L white or opaque buckets.

- Larvae and pupae
  1. Day 1. Remove egg papers/ovistrips. Otherwise, no action.
  2. Day 2. Larvae are ‘split’ or thinned to a density of around 100-200 individuals per rearing bucket. Add ¼ tablet of fish food (Tetramin) to each rearing bucket.
  3. Day 3. In the morning, feed larvae ¼ tablet per bucket. Split the larvae further to a density of approximately 100-150 larvae per rearing bucket and ensure all buckets have food.
  4. Day 4. Feed larvae ½ tablet per container. From this point onwards break up the fish food tablet up and distribute evenly throughout the container.
  5. When feeding larvae, check them first thing in the morning and last thing in the afternoon to ensure larvae are not left without food.
  6. Do not overfeed larvae. Add food when approximately 20% of the tablet from the previous feed is left.
  7. Day 5. Feed *ad libitum*
  8. Day 6. Feed *ad libitum*. If pupae are present, ‘pick’ or remove pupae from the rearing containers using plastic pipettes. Pupae may be kept or killed depending on the requirements of the colony or experiment.
  9. Day 7. No action. (Food should not be required).
  10. Day 8. ‘Bulk’ pupae. Remove all pupae and remaining larvae from the rearing container and place in a plastic 500ml cup. Ensure that the water is the cup is changed to clean water as use of used pan water can lead to bacterial blooms when introduced into the cage that cause high pupal mortality and colony loss. 2-300 pupae can be placed in each cup. Ensure the side of the cup has been roughened (scratched with sandpaper) first to facilitate emergence. Place the cup containing pupae in the adult emergence cage.
  11. Pupae must be removed (either picked or bulked) from rearing containers in < 45 hour intervals (less than every two days) to ensure that adults do not emerge before the pupae are in cages.
